# Combined use of *Enterobacter* sp. MN17 and zeolite reverts the adverse effects of cadmium on growth, physiology and antioxidant activity of *Brassica napus*

**DOI:** 10.1371/journal.pone.0213016

**Published:** 2019-03-13

**Authors:** Zahra Saeed, Muhammad Naveed, Muhammad Imran, Muhammad Asaad Bashir, Annum Sattar, Adnan Mustafa, Azhar Hussain, Minggang Xu

**Affiliations:** 1 Institute of Soil and Environmental Sciences, University of Agriculture Faisalabad, Faisalabad, Pakistan; 2 Soil and Environmental Sciences Division, Nuclear Institute for Agriculture and Biology, Faisalabad, Pakistan; 3 Soil and Water Testing Laboratory, Sheikhupura, Pakistan; 4 National Engineering Laboratory for Improving Quality of Arable Land, Institute of Agricultural Resources and Regional Planning, Chinese Academy of Agricultural Sciences, Beijing, China; 5 Department of Soil Science, University College of Agriculture and Environmental Sciences, The Islamia University of Bahawalpur, Bahawalpur, Pakistan; Estacion Experimental del Zaidin, SPAIN

## Abstract

The objective of the study was to evaluate role of zeolite and *Enterobacter* sp. MN17 on Cd uptake, growth, physiological and biochemical responses of *Brassica napus* on Cd-contaminated soil. A sandy clay loam soil in plastic pots was spiked with Cd (0 and 80 mg kg^-1^) and amended with zeolite (0 and 10 g kg^-1^). Seeds of *B*. *napus* were inoculated with *Enterobacter* sp. MN17. Both inoculated and non-inoculated seeds of *B*. *napus* were sown and plants were harvested after 60 days of growth and data were collected. Although sole application of zeolite and seed inoculation reverted adverse effects of Cd in *B*. *napus* plants, the combined use resulted in even higher growth and physiological responses compared to control plants. The combined use under Cd stress increased plant height, root length, dry biomass of shoot and root up to 32%, 57%, 42% and 64%, respectively compared to control. The different physiological attributes (photosynthetic rate, chlorophyll content, transpiration rate, stomatal conductance) of *B*. *napus* were improved from 6% to 137%. Moreover, combined use of zeolite and seed inoculation on Cd-contaminated soil reduced the stress to plants as antioxidant activities decreased up to 25–64%, however enzyme activities were still higher than plants grown on normal soil. Root and shoot analysis of *B*. *napus* for Cd content depicted that zeolite and bacterium decreased Cd uptake from soil. It is concluded that combined use of zeolite and strain MN17 reduces Cd uptake from soil and improves physiological and biochemical responses of *B*. *napus* which is helpful to alleviate Cd toxicity to plants.

## Introduction

Heavy metal pollution of water and soil resources is an emergent issue of the modern industrial world. Among heavy metals, cadmium (Cd) is considered as one of the most toxic, non-essential element. It enters into soils through anthropogenic activities, atmospheric deposition, volcanic eruptions and weathering of parent materials [1, 2, 3]. The accumulation of Cd in soil deteriorates its quality, affects soil microbes and interferes uptake of plant essential elements [4, 5, 6], thus seriously limiting crop productivity. Cadmium can cause several morpho-physiological and biochemical disorders in plants [7]. Moreover, food chain contamination with Cd threatens human health [8, 9, 10].

This situation demands the development of suitable strategy to remediate Cd-contaminated soils. Various approaches such as physical, chemical and/or biological have been employed to remediate such soils [11, 12, 13]. Physical methods involve replacement of contaminated soils with healthy soils. This technology is large in working volume, costly and is suitable for soil with small area and polluted severely [14]. Among the chemical approaches, different extractants and leachants have been used to remediate Cd-contaminated soils [15]. The leaching and washing of soil for Cd removal has low scope as it causes loss of soil nutrients, ground and surface water pollution. Moreover, success highly depends on soil properties. Hence, it is necessary to develop heavy metal remediation technologies having low cost and environment friendly. Under the scenario, biological remediation approach involving bioremediation, phytoremediation or combination is getting attention worldwide [16]. The main advantages over physico-chemical approaches include cost effectiveness, environment friendly nature, *in situ* remediation and produces little or no secondary residues [17, 18]. The use of metal tolerant microbes and plant species in combination to remediate Cd-contaminated soils is getting popular worldwide. The bacteria associate with plant roots and enhance plant growth through multifarious mechanisms. Such microbes ameliorate heavy metal stress by minimizing their uptake through reduced bioavailability in rhizosphere [19, 20]. Low bioavailability of metals in rhizospher favors better root proliferation and ultimately the plant growth. These metal resistant microbes may immobilize/ modify the availability of metals to plants by synthesis of exopolysaccharides (EPS), siderophores, acidification and/or metal phosphates [21]. The second main factor which may immobilize/adsorb Cd in soil is the use of organic and inorganic amendments. One of the adsorbents is zeolite, a class of alumino-silicate minerals with a negative charge embedded in its porous structure and can adsorb multiple exchangeable cations [22]. The efficiency to adsorb cations depends on exchange capacity and porosity of a particular zeolite [22]. Due to this property such materials are known as molecular sieves and have been studied for Cd removal from aqueous solution/wastewaters [23, 24]. However, there is very limited work on investigating role of zeolite amendment in reducing plant Cd uptake and retrieval of adverse effects on plant growth [24, 25]. Moreover, there are contrasting observations regarding Cd uptake from zeolite amended soils. For instance, Eshghi et al. [25] reported that zeolite reduced Cd accumulation in soybean plant, whereas Mollaei et al. [26] found non-significant increase in Cd uptake in spinach. So, there is need to do further investigations in understanding the role of zeolite on Cd uptake from Cd-contaminated soils. In addition, no information is available regarding physiological and biochemical responses of crops to zeolite application on Cd-contaminated soils. There is also need to investigate the role of using metal tolerant endophytic bacteria and zeolite together on Cd uptake, growth, physiological and biochemical responses of crops grown in Cd-contaminated soil.

It is hypothesized that combined use of metal-tolerant endophytic bacterium, *Enterobacter* sp. MN17, and zeolite may reduce plant Cd uptake from Cd-spiked soil and retrieve Cd-induced growth retardation and physiological disturbances of *B*. *napus*, a commonly used plant species in phytoremediation studies.

## Materials and methods

### Endophytic bacterium and experimental soil

The previously isolated endophytic bacterium, *Enterobacter* sp. MN17 (accession number KT375575), was obtained from the Environmental Sciences Laboratory of Institute of Soil and Environmental Sciences, University of Agriculture Faisalabad, Pakistan. The soil used in study was collected from research field of Institute of Soil and Environmental Sciences, University of Agriculture Faisalabad, Pakistan (31.439052° N, 73.069335° E). A subsample of sieved soil was analyzed for various physico-chemical properties (Table 1). Particle size analysis was done using hydrometer method of Gee and Bauder [27]. The obtained values of soil particles (% clay, % silt, and % sand) were plotted against a textural triangle to get the soil textural class. Saturation percentage (SP) of the soil was determined by following the method of Richard [28];

SP=Wt.ofsoilpaste(g)−Wt.ofdrysoilpaste(g)/Wt.ofovendrysoilpaste(g)

The pH of the soil was measured in a saturated soil paste using pH meter (Kent Eil 7015, UK) [28]. Electrical conductivity of the saturated soil paste extract measured by conductivity meter (Jenway 4070, UK) [28]. Organic matter was estimated by following the method described by Moodie et al. [29]. Total nitrogen was analyzed by Ginning and Hibbard’s method and Kjeldahl’s apparatus was used for digestion [29]. Soil plant available phosphorus content was determined by sodium bicarbonate (NaHCO_3_) extraction method [30]. Soil plant available potassium was estimated through analysis on flame photometer (Jenway PFP7, UK) after extraction by ammonium acetate (NH_4_C_2_H_3_O_2_) [31]. Available Cd concentration in soil was extracted with diethylene triamine penta acetic acid (DTPA), and Cd in the extract was directly measured using atomic absorption spectrometer (PerkinElmer, AAnalyst 100, Waltham, USA).

**Table 1:** Physico-chemical properties of soil.

Characteristics of soils	Units	Values
pH		8.02
Electrical conductivity (EC)	dS m^-1^	1.17
Soil texture		Sandy clay loam
Organic matter	%	0.61
Equivalent calcium carbonate	%	2.47
Total nitrogen	%	0.046
Available phosphorus	mg kg^-1^	3.46
Available potassium	mg kg^-1^	112
Saturation percentage	%	32

### Heavy metal tolerance

Cadmium resistance of the bacterium MN17 was determined following the method of Ahmad et al. [32]. Cadmium nitrate salt was used to develop various levels of Cd (0, 40, 80, 120, 160 and 200 mg mL^-1^) in tryptic soy agar (TSA) plates. The bacterial growth was checked on TSA plates in triplicate. For this purpose, 100 μl inoculum having optical density of 0.50 at 600 nm was poured on to TSA agar plates and the inoculated plates were incubated at 30°C in dark. For strain MN17, the lowest concentration (120 mg mL^-1^) that inhibited visible growth at incubated temperature within 5 days was determined.

### Seed inoculation

The pure culture of strain MN17 was inoculated into 100 mL of sterilized tryptic soy broth (TSB) medium taken in a 250 mL Erlenmeyer flask and placed on an orbital shaker (Firstek Scientific, Tokyo, Japan) at 120 rpm for 24 hours at 28°C. The culture was harvested by centrifugation at 6000 rpm at 21°C for 20 minutes. The population of bacteria was established by turbid metric method and optical density (O.D) was maintained to about 10^8^ cells mL^-1^ broth (OD~660 = 0.08) [33]. Seeds of *B*. *napus* were surface sterilized with 5% sodium hypochlorite solution for 5 minutes and washed thrice with distilled water to remove residues of sterilizing agent [34]. Seeds were inoculated with the broth mixed with 10% sugar solution, peat and clay mixture. Peat to clay ratio was used as 1:1 w/w [35]. The seeds were shaken well till fine coating appeared on seeds. Control was treated with sterilized peat plus clay mixed with sterilized broth and sugar solution. Inoculated seeds were placed over night for drying under laboratory conditions.

### Plant experiment

A pot experiment was conducted in wirehouse to investigate the efficacy of seed inoculation with *Enterobacter* sp. MN17 and zeolite on plant growth, physiology and reduce uptake of Cd in *B*. *napus* in Cd contaminated soil. The soil was air dried, ground and, after passing through a 2 mm sieve, it was thoroughly mixed. Each pot was filled with 8 kg sieved soil. Soil in plastic pots was spiked with Cd (0 and 80 mg kg^-1^) two weeks before seeds sowing, and amended with zeolite (0 and 10 g kg^-1^). To develop desired Cd level in soil, a stock solution of 5000 mg Cd L^-1^ was prepared in distilled water using cadmium nitrate. The volume of distilled water to fully saturate the soil taken in pots was calculated and 640 mg Cd was added into it from stock solution. The seeds of *B*. *napus* were very kindly provided by Ayub Agriculture Research Institute (AARI) Faisalabad, Pakistan. Five seeds (inoculated or non-inoculated) were sown in each pot and the seedlings were thinned to 2 plants per pot on germination. The treatments were applied according to completely randomized design (CRD)-factorial arrangements with three replicates. Recommended doses of nitrogen, phosphorus (P_2_O_5_) and potassium (K_2_O) were applied at the rate of 90, 60 and 75 kg ha^-1^, respectively as urea, single super phosphate (SSP) and sulfate of potash (SOP) during sowing time [36]. The quantity of N added through cadmium nitrate in Cd-spiked soils was subtracted from total N per pot and remaining quantity was added through urea. Soil in pots was maintained at about field capacity during the experiment by daily observations. All the standard agronomic as well as insect and pest management strategies were adopted for this pot experiment.

Plants were harvested after 60 days and plant growth parameters such as plant height, root length, root and shoot dry biomass were recorded. Plant height was taken from the soil surface to the tip of plant by meter rod and average was taken. After harvesting root and shoot samples were placed in an oven for drying at 65°C for 72 hours to determine their dry weights.

### Measurement of physiological and biochemical attributes

#### Physiological attributes

Plant physiological parameters such as photosynthetic rate (A), transpiration rate (E) and stomatal conductance (gs) of the leaves of *B*. *napus* were measured using infra-red gas analyzer (model LCA-4, Germany). Whereas, chlorophyll contents (SPAD) were measured by using chlorophyll meter (SPAD 502, Minolta, Japan).

#### Relative water content and electrolyte leakage

Relative water content (RWC) of plant leaves (one cm^2^ piece without midrib) were determined according to method described by Mayak et al. [37].

RWC(%)=(freshweight−dryweight)/(turgidweight−dryweight)×100

For turgid weight, plant leaves were kept in 100% humidity conditions in the dark at 4°C for 24 hours.

Electrolyte leakage (EL) was determined following the method of Lutts et al. [38]. Plant leaf (one cm^2^ piece without midrib) was placed in test tubes containing 10 mL of distilled water and recorded the electrical conductivity, EC_1_ with EC meter. The tubes were placed on shaker for 2 hours and recorded the EC_2_. Then the tubes were autoclaved at 120°C and after cooling EC_3_ were recorded.

Electrolyteleakage(%)=(EC2−EC1)/(EC3)×100

#### Antioxidant enzymes assay

For antioxidant enzymes measurements, the fresh frozen leaf samples were homogenized and kept in ice cold solution (200 mM potassium phosphate buffer of pH 7 containing 100 mM EDTA). The activities of six antioxidants including catalase (CAT), ascorbate peroxidase (APX), superoxide dismutase (SOD), glutathione reductase (GR), glutathione-S-transferase (GST) and glutathione peroxidase were measured [39–44].

### Measurement of Cd concentration in soil and plant tissues

For determining DTPA extractable Cd content in soils receiving different treatments, 10 g air dried soil sample was suspended in 20 mL DTPA extractant solution, suspension was shaken for 15 min at 200 rpm on reciprocal shaker [45]. Samples were filtered through Whatman No. 42 filter paper. Cadmium in extract was directly measured using atomic absorption spectrometer (PerkinElmer, AAnalyst 100, Waltham, USA). The shoot and root samples were dried in an oven at 65°C to a constant weight, ground in Wiley mill and 0.5 g of ground material was digested using HNO_3_: HCLO_4_ (3:1) mixture. After digestion, the contents in flasks were cooled, filtered properly and stored in washed plastic bottles for subsequent analysis of Cd on atomic absorption spectrometer following the method described by Yong et al. [46].

### Statistical analysis

The collected data were analyzed statistically by employing the Fisher’s analysis of variance technique [47], and treatment’s mean were compared by using LSD test at 5% probability level.

## Results

### Heavy metal tolerance of *Enterobacter* sp. MN17

The tolerance of endophytic bacterium, *Enterobacter* sp. MN17, to Cd was estimated in TSA medium (Table 2). The *Enterobacter* sp. strain MN17 was able to survive and grow well on TSA medium up to 120 mg Cd mL^-1^, however, no bacterial growth was observed at 200 mg Cd mL^-1^.

**Table 2:** Type of growth of *Enterobacter* sp. MN17 on TSA agar plates containing different concentration of cadmium.

Cadmium concentration (mg mL^-1^)	Type of growth on petri-plates
0	Very heavy growth
40	Heavy growth
80	Medium growth
120	Light growth
160	Very light growth
200	No growth

### Plant growth parameters

The individual and combined effect of zeolite and *Enterobacter* sp. MN17 on growth attributes of *B*. *napus* in normal and Cd contaminated soil is shown in Table 3. The plant growth (plant height, root length, shoot dry biomass and root dry biomass) significantly (*p<0*.*05*) decreased in Cd-contaminated soil than normal soil. Application of zeolite and *Enterobacter* sp. MN17 individually as well as in combination retrieved Cd-induced growth suppression of *B*. *napus*. However, better growth retrieval response of plants was observed on their combined application, even better growth was observed than control (no Cd, no zeolite and no inoculation). Zeolite and strain MN17 also significantly (*p<0*.*05*) improved growth on normal soil. The combined application of zeolite and *Enterobacter* sp. MN17 increased plant height up to 29% and 32%, root length up to 54% and 57%, dry biomass of shoot up to 34% and 42%, and dry biomass of root up to 51% and 64% in normal and Cd contaminated soil, respectively. In general, zeolite and bacterium improved the growth more on Cd contaminated soil than normal soil as higher percent increases in different growth attributes were observed.

**Table 3:** Combined impact of zeolite and *Enterobacter* sp. MN17 on growth of *Brassica napus* grown in normal and Cd contaminated soil.

Treatments	Plant height (cm)		Root length (cm)		Shoot dry biomass (g pot^-1^)		Root dry biomass (g pot^-1^)	
	*0 mg Cd kg*^*-1*^	*80 mg Cd kg*^*-1*^	*0 mg Cd kg*^*-1*^	*80 mg Cd kg*^*-1*^	*0 mg Cd kg*^*-1*^	*80 mg Cd kg*^*-1*^	*0 mg Cd kg*^*-1*^	*80 mg Cd kg*^*-1*^
Control	127e	111f	11e	9f	19e	15g	1.2e	0.9f
Zeolite	139 c	124e	13d	11e	20d	18f	1.3d	1.1e
*Enterobacter* sp. MN17	148b	133d	14b	13c	23b	19e	1.6b	1.5c
*Enterobacter* sp. MN17 + Zeolite	163a	147b	17a	14b	25a	22c	1.8a	1.6bc

Data are shown as mean of three replicates, p< 0.05 (probability level)

### Physiological parameters

Like growth attributes, photosynthetic rate (A), transpiration rate (E), stomatal conductance (g_s_) and SPAD chlorophyll content of leaves of *B*. *napus* were modified by Cd stress (Table 4). A decrease from 5.0 to 4.3 μmol CO_2_ m^-2^ s^-1^, 3.2 to 2.3 mmol H_2_O m^-2^ s^-1^, 154 to 125 mmol H_2_O m^-2^ s^-1^ and 36.0 to 32.6 was recorded in *A*, *E*, *g*_*s*_ and SPAD readings, respectively by Cd treatment. However, it was found that zeolite and *Enterobacter* sp. MN17 ameliorated the physiological disturbance caused by Cd treatment. The combined application of zeolite and *Enterobacter* sp. MN17 showed better amelioration in normal as well as Cd contaminated soil compared to their sole application. Up to 174% and 137% increase in photosynthetic rate, 61% and 105% in transpiration rate, 45.7% and 67.2% stomatal conductance and 44.8% and 44% in SPAD chlorophyll contents were observed in normal and Cd-stressed conditions, respectively as compared to their negative controls.

**Table 4:** Combined impact of zeolite and *Enterobacter* sp. MN17on physiological attributes of *Brassica napus* grown in normal and Cd contaminated soil.

Treatments	Photosynthetic rate (A) (μmol CO_2_ m^-2^ s^-1^)		Transpiration rate (E) (mmol H_2_O m^-2^ s^-1^)		Stomatal conductance (gs) (mmol H_2_O m^-2^ s^-1^)		Chlorophyll content (SPAD reading) (mg cm^-2^)	
	*0 mg Cd kg*^*-1*^	*80 mg Cd kg*^*-1*^	*0 mg Cd kg*^*-1*^	*80 mg Cd kg*^*-1*^	*0 mg Cd kg*^*-1*^	*80 mg Cd kg*^*-1*^	*0 mg Cd kg*^*-1*^	*80 mg Cd kg*^*-1*^
Control	5.00e	4.30f	3.2e	2.3f	154f	125g	36e	32.63f
Zeolite	6.80c	5.93d	4.2c	3.7d	201c	175e	41.5d	37.7e
*Enterobacter* sp. MN17	10.10b	7.20c	4.6b	4.3c	214b	191d	44.2c	41.5cd
*Enterobacter* sp. MN17 + Zeolite	13.70a	10.23b	5.2a	4.7b	225a	209b	52.13a	47b

Data are shown as mean of three replicates, p< 0.05 (probability level)

### Water relations

**Fig 1:**
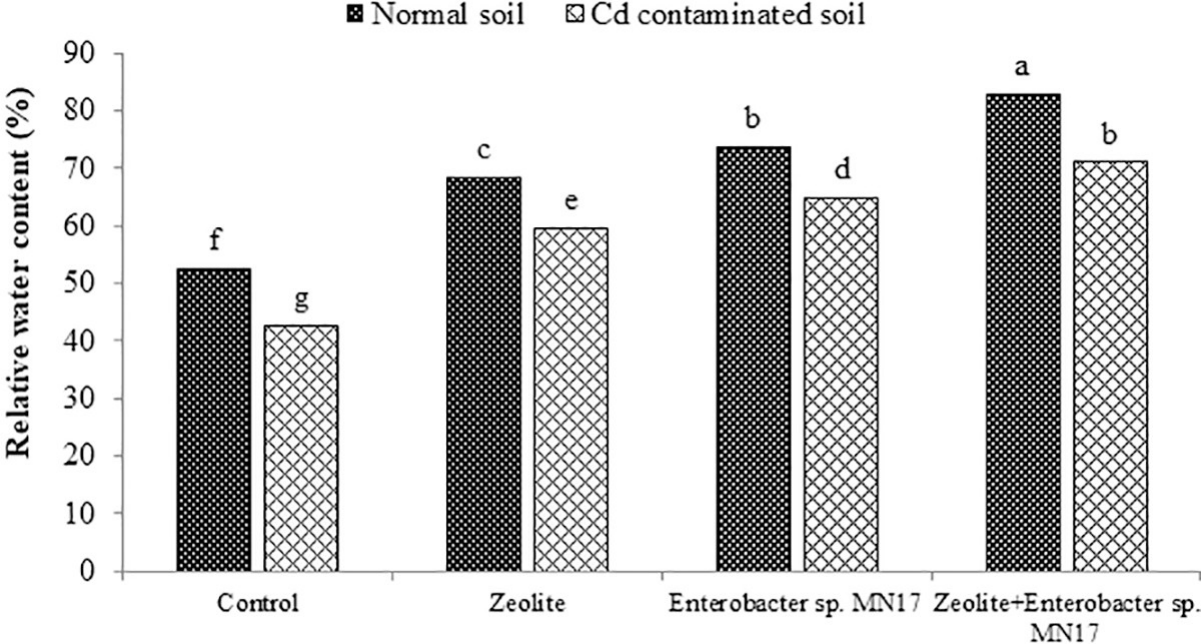
Combined impact of zeolite and *Enterobacter* sp. MN17 on relative water content (%) of *Brassica napus* grown in normal and Cd contaminated soil.

**Fig 2:**
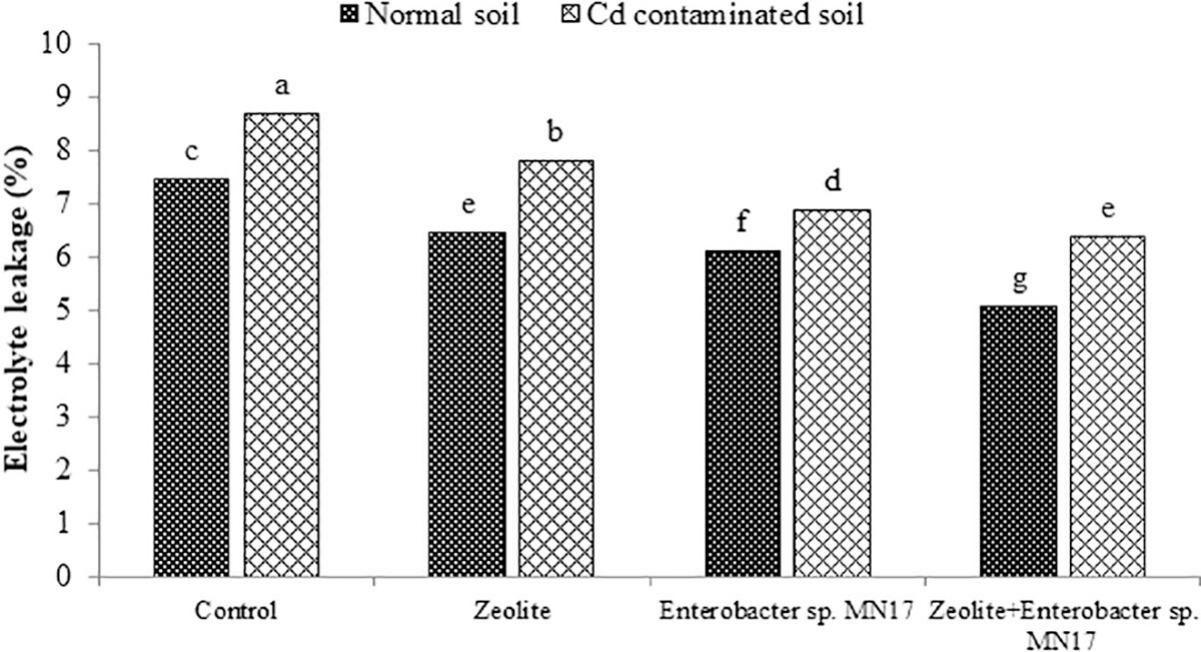
Combined impact of zeolite and *Enterobacter* sp. MN17 on electrolyte leakage (%) of *Brassica napus* grown in normal and Cd contaminated soil.

The RWC of plant leaves and *EL* due to membrane injury significantly (*p<0*.*05*) varied between *B*. *napus* plants grown on normal and Cd contaminated soils. In control plants (no amendment), a decrease in RWC up to 52% and 43% (Fig 1) and increase in *EL* up to 7.5 and 8.7% (Fig 2) was observed in normal and Cd contaminated soil, respectively. Compared to control treatment, the application of zeolite and *Enterobacter* sp. MN17 improved RWC of 59–74%, and decreased EL of 6.9–7.8%, respectively on normal and Cd-contaminated soils. The application of zeolite and *Enterobacter* sp. MN17 improved the RWC to 83.0 and 71.1% and EL to 5.1 and 6.4% in normal and Cd contaminated soil, respectively. The use of zeolite and *Enterobacter* sp. MN17 together produced higher response in terms of RWC and lower in terms of EL in Cd contaminated than normal soil.

### Antioxidant activities

In Cd stress environment, the activity of antioxidants including CAT, APX, SOD, GR, GPX and GST was modified, and it increased from 22.5 to 28.6, 61.1 to 74.2, 168.8 to 226.1, 35.9, 39.4, 0.153 to 0.164 and 16.6 to 20.2 nmol min^-1^ mg^-1^ protein, respectively compared to control (Table 5). The application of zeolite and *Enterobacter* sp. MN17 significantly (*p<0*.*05*) decreased the activity of antioxidants alone and in combination. However, antioxidant activity was further lowered by the combined use of zeolite and *Enterobacter* sp. MN17 in *B*. *napus*. Under Cd stress, the activity of CAT, APX, SOD, GR, GPX and GST decreased up to 25%, 35%, 63%, 33%, 17% and 18%, respectively compared to respective control.

**Table 5:** Combined impact of zeolite and *Enterobacter* sp. MN17 on the activity of antioxidants of *Brassica napus* grown in normal and Cd contaminated soil.

Treatments	Enzyme activity (nmol mint^-1^ mg^-1^ protein)											
	Catalase (CAT)		Ascorbate peroxidase (APX)		Superoxide dismutase (SOD)		Glutathione reductase (GR)		Glutathione peroxidase (GPX)		Glutathione S-transferase (GST)	
	*0 mg*	*80 mg Cd kg*^*-1*^	*0 mg*	*80 mg Cd kg*^*-1*^	*0 mg*	*80 mg Cd kg*^*-1*^	*0 mg*	*80 mg Cd kg*^*-1*^	*0 mg*	*80 mg Cd kg*^*-1*^	*0 mg*	*80 mg Cd kg*^*-1*^
	*Cd kg*^*-1*^		*Cd kg*^*-1*^		*Cd kg*^*-1*^		*Cd kg*^*-1*^		*Cd kg*^*-1*^		*Cd kg*^*-1*^	
Control	22.46b	28.60a	61.11b	74.20a	168.79b	226.05a	35.87b	39.36a	0.153b	0.164a	16.61d	20.17a
Zeolite	22.35b	22.70b	56.74c	61.11b	118.80c	166.65b	26.93d	35.72b	0.147c	0.154b	15.46g	18.48b
*Enterobacter* sp. MN17	22.22b	22.30b	48.01d	56.74c	97.84d	112.26c	25.87d	32.46c	0.137e	0.143d	15.78f	18.14c
*Enterobacter* sp. MN17 + Zeolite	18.13d	21.40c	39.28e	48.01d	70.95f	82.52e	19.41e	26.28d	0.115f	0.135e	13.15h	16.52d

Data are shown as mean of three replicates, p< 0.05 (probability level)

### Cadmium concentration in soil and plant tissues

At harvest Cd was not detected in soil, root and shoot of *B*. *napus*, whereas about 19.7, 24.9 and 39.8 mg kg^-1^ Cd was found in root and shoot of *B*. *napus*, respectively (Table 6). Results showed that in Cd-spiked soil, both zeolite and *Enterobacter* sp. MN17 increased DTPA extractable Cd content in soil. The application of zeolite and *Enterobacter* sp. MN17 increased the Cd concentration from 19.7 to 31.4 and 24.3 mg kg^-1^ Cd, respectively, whereas in soil samples treated with both bacterium and zeolite, Cd concentration was 42.6 mg Cd kg^-1^ soil. However, zeolite and bacterium application reduced uptake of Cd to root and shoot of *B*. *napus*. In control treatment, shoot Cd concentration was 39.8 mg kg^-1^ Cd which decreased to 13.7, 21.2 and 13.4 mg kg^-1^ by the application of zeolite, strain MN17 and zeolite + strain MN17, respectively. In case of Cd concentration in *Brassica napus* roots, content was reduced from 24.9 to 17.3, 21.3 and 13.4 mg Cd kg^-1^ by the application of zeolite, strain MN17 and zeolite + strain MN17, respectively.

**Table 6:** Combined impact of zeolite and *Enterobacter* sp. MN17 on cadmium concentration in soil and *Brassica napus* in normal and Cd contaminated soil.

Treatments	Cd concentration in soil		Cd concentration in root		Cd concentration in shoot	
	(mg kg^-1^)		(mg kg^-1^)		(mg kg^-1^)	
	*0 mg Cd kg*^*-1*^	*80 mg Cd kg*^*-1*^	*0 mg Cd kg*^*-1*^	*80 mg Cd kg*^*-1*^	*0 mg Cd kg*^*-1*^	*80 mg Cd kg*^*-1*^
Control	*ND*	19.7d	*ND*	24.9a	*ND*	39.8a
Zeolite	*ND*	31.4b	*ND*	17.3c	*ND*	26.3c
*Enterobacter* sp. MN17	*ND*	24.3c	*ND*	21.2b	*ND*	31.6b
*Enterobacter* sp. MN17 + Zeolite	*ND*	42.6a	*ND*	13.4d	*ND*	21.1d

ND = Not detected, Data are shown as mean of three replicates, p< 0.05 (probability level)

## Discussion

This study evaluates the potential of amending soil with zeolite, an inorganic adsorbent, and seed inoculation with endophytic metal tolerant bacterium, *Enterobacter* sp. MN17, to mitigate the Cd induced changes in growth, physiology and antioxidant activity of *B*. *napus*. Moreover, potential of zeolite and strain MN17 to immobilize Cd in soil with reduced plant uptake has been investigated.

The strain MN17 was found as Cd tolerant bacterium as it can grow in the presence of Cd up to 120 mg mL^-1^ on TSA medium. This endophytic bacterium is more tolerant to Cd than other Cd-tolerant bacteria, *Pseudomonas Serratia* sp. LRE07, *Pseudomonas aeruginosa* ZM-130, *Micrococcus* sp. MU1, *Klebsiella* sp. BAM1 and *Bacillus* sp. L14 [48–51]. High tolerance to Cd makes this bacterium a suitable candidate to be used on soils contaminated with Cd to induce metal tolerance in crop plants. Results of the greenhouse study depicted that growth of *B*. *napus* was significantly (*p<0*.*05*) reduced on Cd-spiked soil than normal soil (Table 3). These results are in accordance with previous reports on *B*. *napus* grown on Cd-contaminated soils [52–54]. Cadmium reduces growth of plants by interfering with nutrient absorption through roots by occupying exchange sites, reduce microbial growth and activities, production of reactive oxygen species (ROS) and physiological disturbances [55–57, 54]. Along with significant reduction in growth of *B*. *napus*, Cd stress reduced photosynthetic rate, chlorophyll content and gas exchange characteristics in leaves of 60-day old *B*. *napus* plants, whereas electrolyte leakage was increased (Table 4; Fig 2). Cadmium affects rate of photosynthesis by disturbing the function of enzymes involved in the photosynthesis and chlorophyll synthesis [58, 59]. Electrolyte leakage accompanies plant response to stresses and is related to potassium ion efflux from plant cells [60]. The other modification in normal response of *B*. *napus* was increased antioxidant activities (CAT, APX, SOD, GR, GPX and GST) (Table 5). The signals of stress trigger the production of different antioxidants to scavenge ROS [54]. The modification of biochemical response of *B*. *napus* in the presence of Cd has already been observed by others [61, 62]. Moreover, it was found that plants grown on Cd-spiked soils had high Cd content in root and shoot of *B*. *napus* (Table 6). The high uptake of Cd in root and shoot was associated with more availability of Cd in spiked soil.

Application of zeolite improved plant growth, physiological and biochemical responses of *B*. *napus* under Cd stress. There are scientific reports on mitigating Cd-induced growth retardation of soybean and spinach by the application of zeolite [25, 26], however no information is available regarding usefulness of zeolite application on ameliorating Cd-induced growth inhibition of *B*. *napus*. This growth retrieval response of *B*. *napus* to zeolite application might have occurred due to decrease in Cd uptake from the plant rhizosphere. Zeolite is a strong adsorbent of metals and has more binding capacity due to its negatively charged ions [63]. Moreover, absorption and retention capacity for major nutrients released from fertilizer and maintenance of adequate water supply might be the reason behind this increase [25]. Zeolite might also improve *B*. *napus* growth by excluding Cd interferences on absorption of other micronutrients. In contrast to our findings Mollaei et al. [26] observed slight increase in Cd uptake in spinach by zeolite application. We also found that zeolite application improved photosynthetic rate, chlorophyll content, stomatal conductance and transpiration rate in both normal and Cd-contaminated soils (Table 4). Recently, De Smedt et al. [64] also observed beneficial effect of foliar application of zeolites on physiological responses of apple trees. Furthermore, it was found that zeolite application decreased antioxidant activities in *B*. *napus* leaves under Cd stress (Table 5). This shows that zeolite amendment has ameliorated the Cd-stress to plant; probably this occurred due to phenomena of immobilization of Cd in rhizosphere. Nozari et al. [65] observed that zeolite application alleviates water stress-induced antioxidant activities in soybean. However, modifications in physiological responses and antioxidant activities of *B*. *napus* by zeolite application in Cd-contaminated soils have not been studied so far.

Although, toxic effects of Cd on soil micro-biota are well-recognized as Cd negatively affects soil biota and cease their activity [66, 67]. But our findings clearly demonstrated the improvement in growth of *B*. *napus* in pot conditions by seed inoculation with plant growth promoting Cd-tolerant endophytic bacterium (*Enterobacter* sp. MN17) both on normal and Cd-contaminated soils. Plant growth promoting endophytic bacteria have also been tested for improving growth of different crops in Cd-contaminated soils [68, 69]. However, there is no report of inducing Cd-resistance to *B*. *napus* through endophytic bacteria. The bacterium, strain MN17, used in this study carried a number of plant growth promoting traits such as production of auxins, cytokinins, gibberellins, siderophores, ACC-deaminase activity, inorganic P solubilization, N fixation and exopolysaccharide (EPS) production [34, 70]. These traits might have helped the plants to perform better in normal and Cd-contaminated soils. Like zeolite, seed inoculation with *Enterobacter* sp. MN17 reduced the uptake of Cd in *B*. *napus* tissues. The EPS producing biofilm forming bacteria are popular to adsorb metals and reduce their absorption through roots of crop plants [71]. The reduced uptake of Cd might have resulted in better physiological and biochemical response of *B*. *napus*, which ultimately reduced Cd-induced growth retardation. The improvement in physiological attributes might be due to the fact that microbes helped to improve the nutrients uptake which further upsurge the chlorophyll from photo-oxidative destruction [72]. Increased chlorophyll contents in leaves may have a positive effect on plant photosynthesis and growth under Cd stress condition.

The combined use of zeolite and bacterial strain MN17 was found superior in improving growth, physiological and biochemical responses of *B*. *napus* grown on normal and Cd contaminated soils. The response was the additive effect of zeolite and seed inoculation with strain MN17. Zeolite might have immobilized the Cd thus rendering them unavailable for the plant uptake, whereas in addition to immobilizing Cd, bacterium might have increased the availability of mineral nutrients to growing plants and improved the root proliferation. Previously, there is no information available in the literature regarding efficacy of combined application of zeolite and Cd-tolerant endophytic bacterium in inducing Cd-resistance to *B*. *napus*,

## Conclusions

In conclusion, application of zeolite and *Enterobacter* sp. MN17 significantly enhanced the plant growth, improved physiological and biochemical responses of *B*. *napus* in normal and Cd contaminated soils. Moreover, both zeolite amendment and seed inoculation with strain MN17 decreased Cd uptake in root and shoot of *B*. *napus*. However, combined application of zeolite and *Enterobacter* sp. MN17 was found more effective in improving *Brassica napus* plant height, root length, dry biomass of root and shoot, chlorophyll content, photosynthetic rate, transpiration rate and stomatal conductance in Cd contaminated soil. The antioxidant activity of CAT, APX, SOD, GR, GPX and GST were decreased. Likewise, zeolite and strain MN17 together reduced Cd uptake in root and shoot of *B*. *napus* on Cd-contaminated soil.
